# Malignant Glioma in the Cerebellum Presenting as Multiple Small Lesions

**DOI:** 10.1155/2019/6725127

**Published:** 2019-01-06

**Authors:** Takashi Mamiya, Shinji Shimato, Toshihisa Nishizawa, Takashi Yamanouchi, Kojiro Ishikawa, Makoto Ito, Masato Abe, Kyozo Kato

**Affiliations:** ^1^Department of Neurosurgery, Kariya Toyota General Hospital, 5-15 Sumiyoshi-cho, Kariya, Aichi, 448-8505, Japan; ^2^Department of Pathology, Kariya Toyota General Hospital, 5-15 Sumiyoshi-cho, Kariya, Aichi, 448-8505, Japan; ^3^Department of Diagnostic Pathology, School of Medicine Fujita Health University, Toyoake, Aichi, Japan

## Abstract

Malignant glioma, the most common malignant primary brain tumor in adults, usually occurs in supratentorial space as a single mass lesion, and cerebellar location and multiple appearance are uncommon. We report a case of a 69-year-old female with three lesions simultaneously found in the cerebellum on magnetic resonance images (MRIs) after suffering from gait disturbance. Two lesions were around 15 mm in size and the other one was observed as a spotty enhancement. Although MRI findings suggested brain metastases, whole body examinations denied any primary malignancies. Biopsy for one lesion in the cerebellum was performed, which resulted in pathological diagnosis of malignant astrocytoma. The lesions were considered multicentric glioma based on MRI definition. The treatment with temozolomide and whole brain radiation was completed. Although the patient was discharged in an independent state with the shrinkage of the tumors, she unexpectedly died following sudden loss of consciousness from an unknown cause one month after discharge. The coincidence of cerebellar location and multicentricity characterized by smallness is quite rare in glioma patients, and such MRI findings might be misleading for the diagnosis. We describe the details of the case and discuss the pathogenesis of this unique presentation of malignant glioma with the literatures.

## 1. Introduction

Glioma is the most common primary malignant brain tumor comprising approximately 30% of all primary brain tumors [1] and usually arises as a single mass lesion. Multiple lesions are categorized as either multifocal gliomas in which the lesions are connected by T2/FLAIR high signal or multicentric gliomas in which they are not [2, 3]. While 12% to 35% of glioblastomas are multifocal, multicentric glioblastomas are much rarer and only account for 2% to 6% of cases [4]. The common location of gliomas in adults is the cerebral hemispheres, and glioma in the cerebellum is infrequent, accounting for 0.6-3.3% of all gliomas [5–7]. Therefore, multicentric glioma evolving in the cerebellum is thought to be quite rare. As for the diagnosis of multiple gliomas, certain features on magnetic resonance image (MRI) such as variable lesion morphology, mild peritumoral edema, and irregular tumor margins can be helpful, although definite diagnosis required histopathology [8, 9].

In this paper, we report a case of malignant glioma which presented with three lesions in the cerebellum. In particular, all lesions were small and considered multicentric based on MRI definition. The appearance of the lesions could be misleading for making diagnosis and treatment strategy. We describe the characteristics of the case and discuss the pathogenesis of the unique presentation, thereby providing some insights into multicentric glioma in the cerebellum.

## 2. Case Presentation

**Figure 1:**
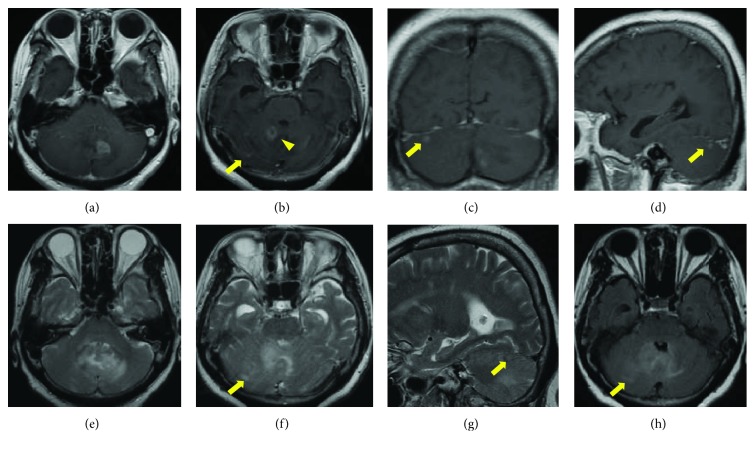
Magnetic resonance images (MRIs) of the patient before biopsy. (a-d) Gd-enhanced T1-weighted images (Gd-T1WI) showing three separate lesions in the cerebellum: the lesion in the left cerebellum near the vermis with relatively regular enhancement (a), the lesion with the similar size in the right cerebellum with ring enhancement (b, arrowhead), and the tiny lesion in right cerebellum located far from two lesions (b, c, d, arrow). T2-weighted images (T2WI) showing peritumoral edema around two lesions (e, f, g), but no edema around the tiny lesion (f, arrow). FLAIR showing no clear connection of the tiny lesion to other two lesions (h, arrow).

A 69-year-old woman, with a history of medication for hypertension, was referred to our hospital because of gait disturbance. Head MRI revealed three separate lesions in the cerebellum: the largest lesion of approximately 15 mm in diameter in the left cerebellum near the vermis with relatively regular enhancement (Figure 1(a)), the lesion of slightly smaller size in the right cerebellum with ring enhancement (Figure 1(b)), and the tiny lesion in the upper right cerebellum located far from the two lesions (Figures 1(b)–1(d)). The smallest lesion was not connected to any other lesions on T2/FLAIR (Figures 1(e)–1(h)).

**Figure 2:**
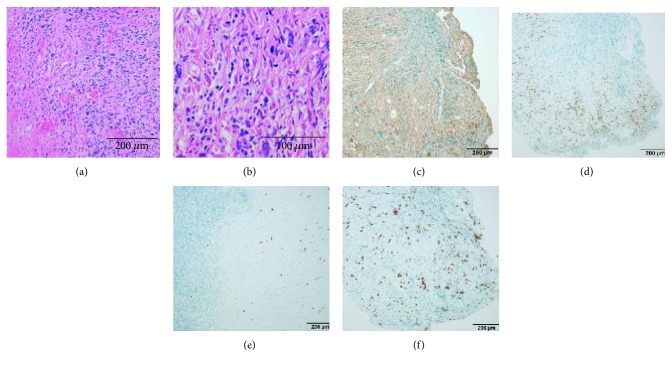
Photomicrographs displaying pathognomonic histopathological features of malignant glioma. (a) H&E staining showed tumor cells with eosinophilic cytoplasm and pleomorphism characterized by dense proliferation and diffuse infiltration in the granular cell layer. (b) Nuclear pleomorphism and mitotic figures were observed, but no microvascular proliferation and micronecrosis were detected. (c) Immunohistochemical staining showed positivity for GFAP. (d) The majority of the tumor cells are positive for p53. (e) P53 staining also revealed infiltrating tumor cells in the granular layer of the cerebellum away from the area of dense tumor cells. (f) The MIB-1 labeling index was 21.3%.

We suspected these lesions were metastatic tumors and performed thorough examination of whole body, which resulted in negative for any primary lesions. Because all lesions were small and debulking surgery was unnecessary, we decided to perform biopsy surgery targeting at the lesion near the vermis. We underwent needle biopsy under the guidance of navigation system, and postoperative course was uneventful. Histopathological examination revealed tumor cells with eosinophilic cytoplasm and pleomorphism, which were characterized by dense proliferation and diffuse infiltration in the granular cell layer of the cerebellum (Figure 2(a)). Nuclear pleomorphism and mitotic figures were observed, but microvascular proliferation and micronecrosis were not detected (Figure 2(b)). Immunohistochemistry revealed the tumor cells were diffusely positive for glial fibrillary acidic protein (GFAP) (Figure 2(c)) and positive for p53 in large part (Figure 2(d)). In particular, p53 clearly showed infiltrating tumor cells at distant area (Figure 2(e)). IDH1 and H3K27M were negative. MIB-1 labeling index was 21.3% (Figure 2(f)). These findings were consistent with WHO grade III anaplastic astrocytoma.

For molecular genetic characteristics, DNA was extracted from frozen tumor tissue. IDH gene was analyzed by direct sequencing, and allelic status of 1p/19q, EGFR, PDGFA, and PTEN was analyzed by multiplex ligation-dependent probe amplification (MLPA) method using SALSA MLPA kit P089 and P105 in accordance with the manufacturer's protocol (HRC Holland, Amsterdam, the Netherlands) [10]. These analyses revealed that the tumor had no allelic loss of 1p/19q, no mutation of IDH, no loss of PTEN, but had the amplification of EGFR and PDGFA.

**Figure 3:**
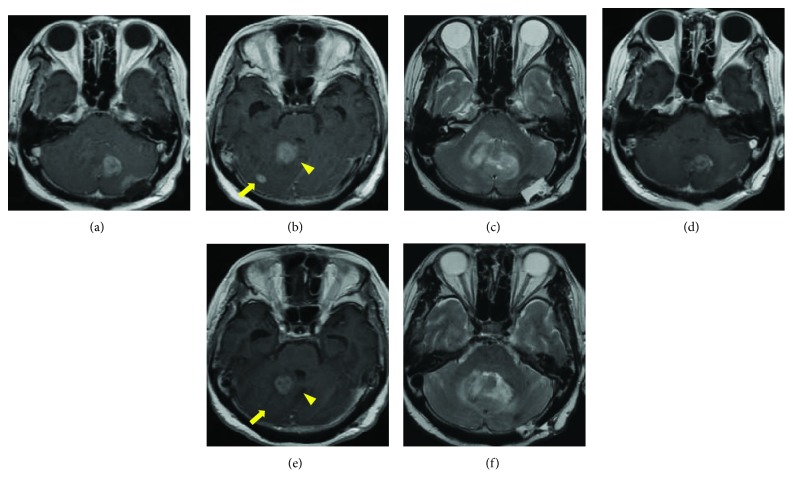
MRIs of the patient after biopsy. (a, b, c) MRIs taken at the midpoint of the treatment with chemoradiotherapy. The enlargement of all lesions and the worsening of the peritumoral edema were observed. (d, e, f) MRIs taken at discharge. Shrinkage of all the tumors and the improvement of the peritumoral edema were observed.

The treatment with whole skull radiation (60 Gy) concomitant with temozolomide was administered and completed without any major side effects. Although the lesions were enlarged during the treatment (Figures 3(a)–3(c)), the patient was discharged in an independent state; the patient was unexpectedly transferred to our hospital due to sudden loss of consciousness and was in the state of cardiopulmonary arrest on arrival. She died shortly despite all possible procedures. CT scans taken immediately after her death revealed no apparent changes in the intracranial area including the cerebellum, thereby denying the correlation of cerebellar glioma with her sudden loss of consciousness. Nothing was found in the whole body that could explain the causes of her death, either. The autopsy was not performed because the consent was not obtained from her family.

## 3. Discussion

In this case, malignant glioma should be taken into account as a differential diagnosis because malignant gliomas can be multicentric and/or multifocal in the posterior fossa [9, 11]. However, we assumed that metastatic brain tumors would be the most possible particularly because all lesions were small. In the literature, there is one case of multiple cerebellar glioma mimicking brain metastasis reported by Walter et al. [12]. In their case, a 69-year-old female case of glioma showed three separate lesions in the cerebellum with one lesion more than 30 mm in diameter and the other two lesions smaller than 11 mm in diameter. While they mentioned that these lesions were initially considered most suspicious to be metastatic brain tumors, two small lesions could be regarded as satellite lesions of the largest lesion, which are sometimes observed in GBM [13]. On the other hand, all lesions in our case were small and appear more confusing for the diagnosis.

Although we successfully made the pathological diagnosis for this patient, the procedure for biopsy for this type of small lesions appeared more troublesome than usual and failure of sampling tumor tissue could happen. Therefore, the diagnosis as brain metastasis might be justified without pathology in such patients even in the situation with no known primary neoplasm, based on the facts that up to 15% of all patients with brain metastases have no clearly detected primary site [14] and that metastasis from extracranial primary tumors is the most common diagnosis associated with intra-axial posterior fossa tumor in adults [15, 16]. In that context, the evidence that glioma can occur in the cerebellum with multiple small lesions, shown by this paper for the first time in the literature, poses an important insight for differential diagnosis, and the necessity of histopathological verification should be underscored.

The mechanism of multiple gliomas has been an intriguing topic and some hypotheses are proposed for explaining the multicentricity of glioma [4]. There might have been a spatial continuity between different foci and an underlying invasion of the whole central nervous system by lower grade cells not visible on the MRI and whose malignant transformation would occur at separate points simultaneously. Another possibility would be that a primary high-grade glioma may spread through cerebrospinal fluid or the white matter tracts to other location [17]. In this regard, while the evidence of malignancy in cerebrospinal fluid was absent in our patient, the specimen from biopsy had an interesting finding that tumor cells were observed sparsely in the area far from tumor cells (Figure 2(e)). This might suggest that the tumor cells had a remarkable capacity of migration, thereby spreading tumor cells could have happened to form the masses at distant sites. Regarding this point, the subventricular zone harbors cell with proliferative potential and has been described as an area which can result in multiple and multicentric GBM, with the expression of metalloproteinase and mesenchymal markers [18, 19]. In our patient, the proximity of one lesion to forth ventricle might support this possibility.

Genetically, the tumor had the amplification of EGFR and PDGFA, no loss of PTEN, and no mutation of IDH, which suggested primary glioblastoma based on the recent genetic investigations [20]. The relationship of genetic status to multicentricity in glioma has been an unknown topic, to our knowledge, but it would be an interesting question for the future, and our report could provide one suggestive data.

Currently, there are no clear guidelines regarding the optimal management of multiple glioma [8], and the role of surgery remains controversial [7, 8]. In our case, aggressive resection apparently should not be indicated because of the location and the size, and biopsy followed by the treatment with temozolomide and radiation would be the most reasonable strategy. While the treatment appeared effective in a short period, the actual prognosis of the glioma in this patient remains unknown because of the unexpected death. Multiple GBM have been associated with a worse prognosis than solitary GBM [8, 15, 16], and the optimal treatment strategy for better prognosis should be pursued for this type of glioma.

We presented the characteristics of a rare case of multicentric glioma in the cerebellum. This report could provide some supplemental suggestions for the differential diagnosis in the patients with small multiple lesions in the cerebellum. The elucidation of the pathogenesis of multiple glioma and the development of the optimal treatment strategy would be the important challenges in the future.

## References

[1] Porter K. R., McCarthy B. J., Freels S., Kim Y., Davis F. G. (2010). Prevalence estimates for primary brain tumors in the United States by age, gender, behavior, and histology. *Neuro-Oncology*.

[2] Batzdorf U., Malamud N. (1963). The problem of multicentric gliomas. *Journal of Neurosurgery*.

[3] Zamponi N., Rychlicki F., Ducati A., Regnicolo L., Salvolini U., Ricciuti R. A. (2001). Multicentric glioma with unusual clinical presentation. *Child's Nervous System*.

[4] Picart T., Le Corre M., Chan-Seng E., Cochereau J., Duffau H. (2018). The enigma of multicentric glioblastoma: physiopathogenic hypothesis and discussion about two cases. *British Journal of Neurosurgery*.

[5] Adams H., Chaichana K. L., Avendano J., Liu B., Raza S. M., Quinones-Hinojosa A. (2013). Adult cerebellar glioblastoma: understanding survival and prognostic factors using a population-based database from 1973 to 2009. *World Neurosurgery*.

[6] Jeswani S., Nuno M., Folkerts V., Mukherjee D., Black K. L., Patil C. G. (2013). Comparison of survival between cerebellar and supratentorial glioblastoma patients: surveillance, epidemiology, and end results (SEER) analysis. *Neurosurgery*.

[7] McGirt M. J., Chaichana K. L., Gathinji M. (2009). Independent association of extent of resection with survival in patients with malignant brain astrocytoma. *Journal of Neurosurgery*.

[8] Hassaneen W., Levine N. B., Suki D. (2011). Multiple craniotomies in the management of multifocal and multicentric glioblastoma. Clinical article. *Journal of Neurosurgery*.

[9] Kuroiwa T., Numaguchi Y., Rothman M. I. (1995). Posterior fossa glioblastoma multiforme: MR findings. *AJNR. American Journal of Neuroradiology*.

[10] Motomura K., Natsume A., Kishida Y. (2011). Benefits of interferon-*β* and temozolomide combination therapy for newly diagnosed primary glioblastoma with the unmethylated MGMT promoter: a multicenter study. *Cancer*.

[11] Utsuki S., Oka H., Miyajima Y., Kijima C., Yasui Y., Fujii K. (2012). Adult cerebellar glioblastoma cases have different characteristics from supratentorial glioblastoma. *Brain Tumor Pathology*.

[12] Walter J., Koch A., Herbold C. (2013). Multifocal glioblastoma multiforme in the posterior fossa mimicking cerebral metastases: case presentation and review of the current literature. *Journal of Neurological Surgery Part A: Central European Neurosurgery*.

[13] Pope W. B., Sayre J., Perlina A., Villablanca J. P., Mischel P. S., Cloughesy T. F. (2005). MR imaging correlates of survival in patients with high-grade gliomas. *AJNR. American Journal of Neuroradiology*.

[14] Han H. J., Chang W. S., Jung H. H., Park Y. G., Kim H. Y., Chang J. H. (2016). Optimal treatment decision for brain metastases of unknown primary origin: the role and timing of radiosurgery. *Brain Tumor Research and Treatment*.

[15] Levine S. A., McKeever P. E., Greenberg H. S. (1987). Primary cerebellar glioblastoma multiforme. *Journal of Neuro-Oncology*.

[16] Stupp R., Hegi M. E., Mason W. P. (2009). Effects of radiotherapy with concomitant and adjuvant temozolomide versus radiotherapy alone on survival in glioblastoma in a randomised phase III study: 5-year analysis of the EORTC-NCIC trial. *The Lancet Oncology*.

[17] Rees J. H., Smirniotopoulos J. G., Jones R. V., Wong K. (1996). Glioblastoma multiforme: radiologic-pathologic correlation. *Radiographics*.

[18] Shakur S. F., Bit-Ivan E., Watkin W. G., Merrell R. T., Farhat H. I. (2013). Multifocal and multicentric glioblastoma with leptomeningeal gliomatosis: a case report and review of the literature. *Case Reports in Medicine*.

[19] Showalter T. N., Andrel J., Andrews D. W., Curran W. J., Daskalakis C., Werner-Wasik M. (2007). Multifocal glioblastoma multiforme: prognostic factors and patterns of progression. *International Journal of Radiation Oncology, Biology, Physics*.

[20] Louis D. N., Ohgaki H., Wiestler O. D. (2016). *WHO Classification of Tumors of the central Nervous System*.

